# Voting with their feet - predictors of discharge against medical advice in Aboriginal and non-Aboriginal ischaemic heart disease inpatients in Western Australia: an analytic study using data linkage

**DOI:** 10.1186/1472-6963-13-330

**Published:** 2013-08-20

**Authors:** Judith M Katzenellenbogen, Frank M Sanfilippo, Michael ST Hobbs, Matthew W Knuiman, Dawn Bessarab, Angela Durey, Sandra C Thompson

**Affiliations:** 1 Combined Universities Centre for Rural Health, University of Western Australia, Geraldton, Australia; 2 School of Population Health, University of Western Australia, Perth, Australia; 3 Curtin Health Innovation Research Institute, Curtin University, Perth, Australia

**Keywords:** Discharge against medical advice, Aboriginal health, Ischaemic Heart Disease, Linked data, Australia

## Abstract

**Background:**

Discharge Against Medical Advice (DAMA) from hospital is associated with adverse outcomes and is considered an indicator of the responsiveness of hospitals to the needs of Aboriginal and Torres Strait Islander Australians, the indigenous people of Australia. We investigated demographic and clinical factors that predict DAMA in patients experiencing their first-ever inpatient admission for ischaemic heart disease (IHD). The study focuses particularly on the differences in the risk of DAMA in Aboriginal and non-Aboriginal patients while also investigating other factors in their own right.

**Methods:**

A cross-sectional analytical study was undertaken using linked hospital and mortality data with complete coverage of Western Australia. Participants included all first-ever IHD inpatients (aged 25–79 years) admitted between 2005 and 2009, selected after a 15-year clearance period and who were discharged alive. The main outcome measure was DAMA as reflected in the hospital record.

Multiple logistic regression was used to determine disparities in DAMA between Aboriginal and non-Aboriginal patients, adjusting for a range of demographic and clinical factors, including comorbidity based on 5-year hospitalization history. A series of additional models were run on subgroups of the cohort to refine the analysis. Ethics approval was granted by the WA Human Research and the WA Aboriginal Health Ethics Committees.

**Results:**

Aboriginal patients comprised 4.3% of the cohort of 37,304 IHD patients and 23% of the 224 DAMAs. Emergency admission (OR=5.9, 95% CI 2.9-12.2), alcohol admission history (alcohol-related OR=2.9, 95% CI 2.0-4.2) and Aboriginality (OR 2.3, 95% CI 1.5-3.5) were the strongest predictors of DAMA in the multivariate model. Patients living in rural areas while attending non-metropolitan hospitals had a 50% higher risk of DAMA than those living and hospitalised in metropolitan areas. There was consistency in the ORs for Aboriginality in the different multivariate models using restricted sub-cohorts and different Aboriginal identifiers. Sex, IHD diagnosis type and co-morbidity scores imparted different risks in Aboriginal versus non-Aboriginal patients.

**Conclusions:**

Understanding the risks and reasons for DAMA is important for health system policy and proactive management of those at risk of DAMA. Improving care to prevent DAMA should target unplanned admissions, rural hospitals and young men, Aboriginal people and those with alcohol and mental health comorbidities.

## Background

Discharge Against Medical Advice (DAMA) from hospital, while relatively uncommon, is associated with re-admission, [1-4] increased morbidity [4] and mortality [2,3] and increased health system costs [5]. International literature consistently reports that risk factors for DAMA include male gender, young adulthood, alcohol abuse with and without psychiatric illness, and social disadvantage [6]. Australian studies [7-9] report particularly high proportions of DAMA in patients admitted for mental and behavioural disorders, and injury and poisoning. Aboriginal and Torres Strait Islander (hereafter Aboriginal) people who comprise 2.5% of the population are substantially over-represented in DAMA, with a fifth of the 65,065 hospital DAMAs between July 2006 and June 2008 occurring among Aboriginal patients [10]. Aboriginal patients had almost 6 times higher age-standardised risk of DAMA than non-Aboriginal patients, while population-based age-standardised rates were almost 12 times higher [10]. A recent national Australian report showed that Aboriginal to non-Aboriginal disparities in DAMA risks following any admission were highest in the Northern Territory, South Australia and Western Australia (WA) [10], and that the disparity increased with remoteness of residence. DAMA rates are now considered an indicator of the responsiveness of hospitals to Aboriginal needs and of the quality of care they receive [11].

High cardiovascular morbidity and mortality rates in Aboriginal people contribute 23% to the Aboriginal health gap [12]. The large disparities are exemplified by high incidence rate ratios for myocardial infarction (MI), particular among men and women 25–54 years [13] and significantly poorer 2-year outcomes post-MI [14]. Improving outcomes for Aboriginal cardiac patients is a priority for the health sector, with high quality hospital care an integral part of the continuum of care [7]. To date, most Australian research into DAMA has focused either on emergency departments [15] or on all-cause or broad disease groups [10] rather than specific clinical subgroups. Investigation of broad disease groups (for example, by International Classification of Diseases-10 chapter) limits the ability to elucidate the extent to which other factors, including demographic and clinical factors, explain the higher Aboriginal risk of DAMA. Additionally, there is a dearth of reporting of DAMA and its determinants in the Australian situation in the peer reviewed literature.

Given the high burden of heart disease in the Aboriginal population, the high DAMA disparities in WA and the availability of state-wide linked health data allowing admission histories to be obtained, [16] this study aimed to investigate demographic and clinical factors that predict DAMA in patients experiencing their first-ever inpatient admission for ischaemic heart disease (IHD). The study focuses particularly on the differences in the risk of DAMA in Aboriginal and non-Aboriginal patients while also investigating other factors in their own right.

## Methods

A person-linked file of all admissions to any WA hospital in 1985–2008 with a discharge diagnosis of IHD (International Classification of Diseases-10 Australian Modification (ICD-10-AM) codes I20-I25) was constructed using the WA Data Linkage System [16]. These data were used to assemble an historical cohort (age range 25–79 years) of all first-ever admissions for IHD in 2000–08, based on a principal discharge diagnosis of IHD after applying a 15-year exclusion period for previous IHD hospitalisation. The linked file consisted of the incident IHD admission (including booked and emergency admissions) and all associated/contiguous hospital transfers. This very specific (‘clean’) cohort was selected to ensure that the patient group was relatively homogeneous with respect to diagnosis (all IHD), primacy of the diagnosis relative to other diagnoses coded for that admission (principal diagnosis only) and history of previous admission for IHD (first-ever), even though it reduced the number of DAMA events. Additional analyses were conducted on subgroups of the cohort to further refine the analyses (see restriction of statistical models under Statistical Analysis).

Characteristics of the cohort were identified, with discharge diagnoses categorised as myocardial infarction (MI, ICD-10-AM code I21 or I22), unstable angina (UA, I20.0) or other IHD (all codes I20-I25 that were not MI or UA). To account for the 5-20% under-identification of Aboriginality in administrative health data, [17] any person identified as Aboriginal on at least 25% of their hospital admissions since 1985 was considered to be Aboriginal. Remoteness of residence was categorised using the postcode-based Accessibility/Remoteness Index of Australia plus (ARIA+) as a starting point. For descriptive analyses, the five-category ARIA+ strata [18] were consolidated into three groups (metropolitan, regional and very remote). ‘Metropolitan’ included all Statistical Local Authorities falling into the WA Metropolitan Health Region (as defined by the WA Department of Health [19], including some usually categorised by ARIA+ as Inner Regional postcodes. Hospitals were categorised as private, district, regional, teaching and non-teaching metropolitan. These two variables were later combined and simplified for the regression analysis due to the close relationship between the hospital and residential location. (see Statistical analysis) In the absence of individual-level data on socioeconomic status (SES), the Index of Relative Socio-economic Disadvantage [20] (area-level score of SES derived from census variables) was used to categorise cases into SES quartiles based on postcodes.

Prior co-morbidity was determined from each patient’s hospital records dating back five years from the incident admission. The Charlson Co-morbidity Index, an instrument used for risk adjustment in outcomes research, was calculated using the Dartmouth-Manitoba ICD code assignments [21] and any history of admissions in WA for mental health and/or alcohol-related conditions was noted. A 10-year history of DAMA and DAMA during the incident IHD episode were based on the discharge type variable in the data set. The type of admission (emergency/booked) was also recorded.

### Statistical analysis

The analysis was conducted in SAS version 9.3. Baseline demographic and co-morbidity characteristics were summarised, and univariate and multivariate logistic regression models were used to determine predictors of DAMA. Baseline demographic (age, sex, Aboriginality, social disadvantage, and residential location), clinical (type of IHD diagnosis, Charlson co-morbidity index, history of mental health or alcohol-related admission) and admission-related (hospital type, emergency or booked admission, calendar period) variables were included in the models, allowing the calculation of adjusted odds ratios taking these variables into account. Combined residential/hospital location was categorised as metropolitan hospital and residence; rural hospital and residence; metropolitan hospital and rural residence; rural hospital and metropolitan residence; and private hospital (irrespective of residence). All non-metropolitan hospital and residence categories defined in the Methods section were considered ‘rural’. Besides using the Aboriginal definition described earlier, separate models used two different methods of identifying Aboriginal status: (i) Aboriginal on all hospital admissions in 1980–2008; and (ii) Aboriginal on index admission only.

The consistency of the risk factors for DAMA was further evaluated by applying the models to different sub-cohorts of the full cohort. In the first instance, the cohort was restricted to patients were emergency admissions due to the fact that DAMA was so rare among booked admissions. Second, a model was fitted to patients who were publicly funded at their discharge hospital due to the overwhelming protective factor of attending a private hospital. Because a history of DAMA has been shown to be strongly predictive of subsequent DAMA, another model was fitted on patients who had not had a DAMA in the previous 10 years. To check that receipt of a procedure might influence the odds of DAMA, we also restricted the cohort to those who did not have coronary artery revascularisation procedures in their incident episode (these procedures are only available in 7 metropolitan hospitals). Logistic regression modeling was also applied separately to Aboriginal and non-Aboriginal patients to investigate risk factors for DAMA stratified by Aboriginal status.

Ethical approval was obtained from the WA Human Research Ethics Committee, the University of WA Human Research Ethics Committee and the WA Aboriginal Human Ethics Committee.

## Results

Between 2000 and 2008, 37,304 people (35,702 non-Aboriginal, 1602 Aboriginal) were admitted with a first-ever IHD hospital diagnosis and discharged alive from WA hospitals following their episode of care, including 224 (172 non-Aboriginal, 52 Aboriginal) DAMAs (Table 1). Thus, Aboriginal patients comprised 4.3% of the cohort and 23% of the DAMAs. Compared with non-Aboriginal patients, a greater proportion of Aboriginal patients were female, lived in rural and socially disadvantaged areas and had an emergency admission (Table 1). Aboriginal patients were more likely to have an IHD diagnosis of MI and have a history of alcohol-related admissions than non-Aboriginal, and 15.6% of the Aboriginal IHD cohort had a DAMA in the previous 10 years compared with 0.9% of non-Aboriginal patients (Table 1).

**Table 1:** Characteristics of first IHD cases (principal diagnosis) in Western Australia 2000–2008, by Aboriginality

		**Non-aboriginal**		**Aboriginal**		
		**Number**	**(%)**	**Number**	**(%)**	**p-value**
Total patients		35,702		1602		
DAMA	in incident admission	172	(0.5)	52	(3.2)	p<0.0001
Sex	Male	24517	(68.7)	881	(55.0)	p<0.0001
	Female	11185	(31.3)	721	(45.0)	
Age	mean (years)	61.9		49.5		p<0.0001
Residence	Metropolitan	28362	(79.4)	515	(32.1)	p<0.0001
	Regional	6130	(17.2)	383	(23.9)	
	Very Remote	1185	(3.3)	702	(43.8)	
	Unknown	25	(0.1)	2	(0.1)	
Social disadvantage	1 (High disadvantage)	7423	(20.8)	857	(53.5)	p<0.0001
	2	9552	(26.8)	413	(25.8)	
	3	6949	(19.5)	207	(12.9)	
	4 (Low disadvantage)	11518	(32.3)	96	(6)	
	Unknown	260	(0.7)	29	(1.8)	
Hospital type	Metro Teaching	19161	(53.7)	1036	(64.7)	p<0.0001
	Metro Non-Teaching	1653	(4.6)	53	(3.3)	
	Regional	1386	(3.9)	231	(14.4)	
	District	1102	(3.1)	255	(15.9)	
	Private (Not public funded)	12400	(34.7)	27	(1.7)	
Admission type	Booked	14381	(40.3)	263	(16.4)	p<0.0001
	Emergency	21321	(59.7)	1339	(83.6)	
IHD diagnosis	MI	11782	(33)	655	(40.9)	p<0.0001
	UA	7634	(21.4)	377	(23.5)	
	Other IHD	16286	(45.6)	570	(35.6)	
Revascularisation in episode	Yes	25893	(72.5)	1241	(77.5)	p<0.0001
	No	9809	(27.5)	361	(22.5)	
Charlson score	Mean	1.3		2.3		p<0.0001
5-year history of alcohol/mental health admission	Neither	33111	(92.7)	1117	(69.7)	p<0.0001
	Alcohol	1121	(3.1)	351	(21.9)	
	Mental Health	1141	(3.2)	60	(3.7)	
	Both	329	(0.9)	74	(4.6)	
Length of stay	mean (days)	3.9		6.1		P<0.0001
DAMA in previous 10 years	Yes	334	(0.9)	250	(15.6)	p<0.0001
	No	35368	(99.1)	1352	(84.4)	

**Figure 1:**
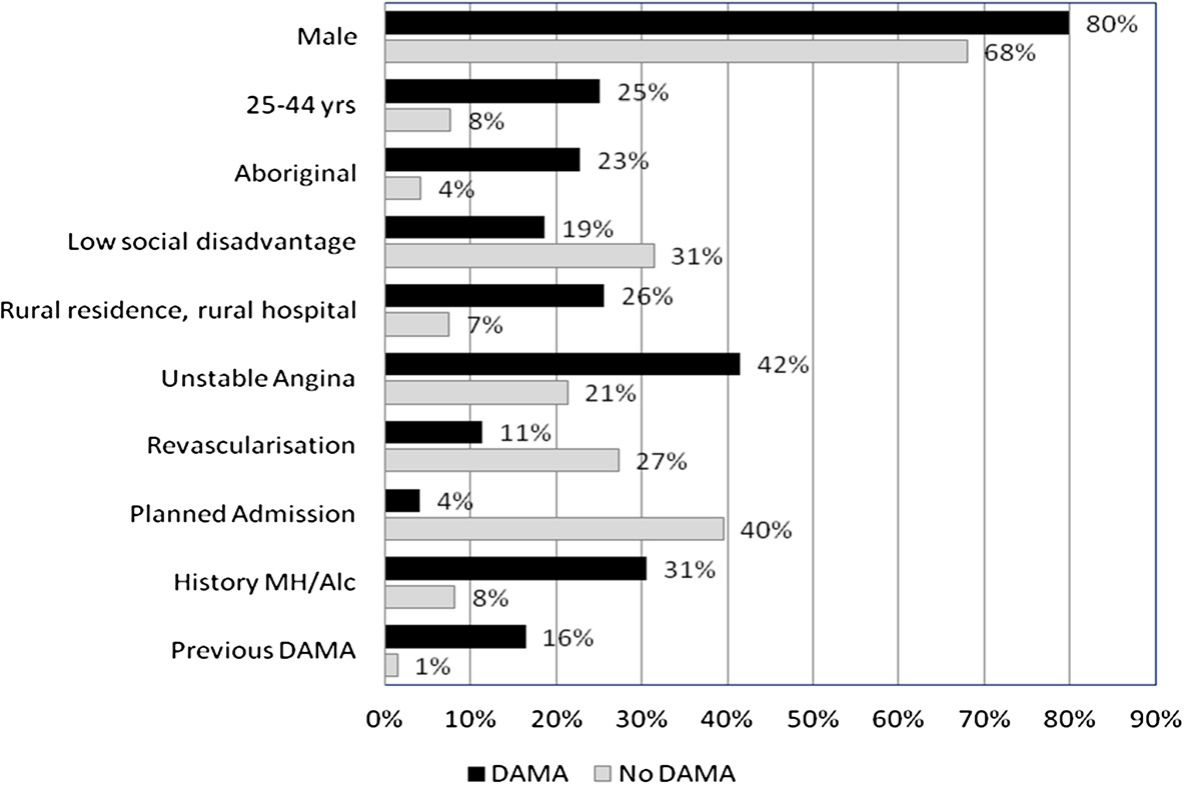
**Comparison of the characteristics of IHD patients who did and did not discharge against medical advice (DAMA), WA 2000–2008.** History MH/Alc: Admission for mental health or alcohol-related disorder in the previous 5 years. All variables in the table differed significantly between cases that did and did not DAMA.

Although the average length of stay was similar in DAMA (3.6 days) and non-DAMA (3.8) patients, DAMA cases differed significantly from non-DAMA patients in many other attributes, being more likely to be male, young, Aboriginal and rural residents (Figure 1). Patients were also more likely to DAMA if they were admitted as an emergency, had a diagnosis of UA and a 10-year history of mental health or alcohol-related admission(s). All these factors were significant in the univariate regression model and attenuated to varying degrees after adjustment for other factors in the multivariate model (Table 2). The strongest positive associations for the whole cohort based on unadjusted odds ratios (OR) were with emergency admissions (OR=14.9, 95% CI 7.6-29.1), Aboriginality (OR=8.4, 95% CI 6.0-11.7) and 5-year history of alcohol-related admissions (OR=7.3, 95% CI 5.1-10.3) while the strongest factor protective against DAMA was admission to a private hospital (OR=0.08, 95% CI 0.04-0.16) (Table 2).

**Table 2:** Odds ratios (OR) for discharge against medical advice during first-ever hospital episode for ischaemic heart disease for full and restricted sub-cohorts, Western Australia 2000-2008

		**Full cohort N=37,015**				**Emergency only N=22,459**		**Publicly funded N=25,664**		**No prior DAMA N=36,447**		**No revascularisation N=26,920**	
		**Events=219 R-Square=0.163**				**Events=210 R-Square=0.111**		**Events=212 R-Square=0.123**		**Events=183 R-Square=0.148**		**Events=194 R-Square=0.169**	
		**Crude OR (95%)**		**Adj OR (95% CI)**		**Adj OR (95% CI)**		**Adj OR (95% CI)**		**Adj OR (95% CI)**		**Adj OR (95% CI)**	
**Aboriginality**	**Aboriginal**	**8.41**	**(6.03 - 11.7)**	**2.32**	**(1.54 - 3.48)**	**2.42**	**(1.60 - 3.66)**	**2.34**	**(1.55 - 3.52)**	**1.98**	**(1.24 - 3.18)**	**2.24**	**(1.44 - 3.47)**
	**non-Aboriginal**	1.00		1.00		1.00		1.00		1.00		1.00	
**Sex**	**Male**	**1.90**	**(1.35 - 2.67)**	**2.14**	**(1.52 - 3.02)**	**2.19**	**(1.54 - 3.11)**	**2.18**	**(1.53 - 3.1)**	**2.15**	**(1.47 - 3.15)**	**2.25**	**(1.58 - 3.21)**
	**Female**	1.00		1.00		1.00		1.00		1.00		1.00	
**Age**	**(years)**	**0.94**	**(0.93 - 0.95)**	**0.97**	**(0.95 - 0.98)**	**0.97**	**(0.96 - 0.98)**	**0.97**	**(0.95 - 0.98)**	**0.96**	**(0.95 - 0.98)**	**0.96**	**(0.95 - 0.98)**
**Soc Disadv**^**a**^	**1 (high)**	**2.46**	**(1.67 - 3.63)**	1.25	(0.82 - 1.89)	1.17	(0.76 - 1.78)	1.26	(0.82 - 1.93)	1.37	(0.89 - 2.12)	1.36	(0.87 - 2.13)
	**2**	1.42	(0.94 - 2.15)	0.91	(0.59 - 1.41)	0.88	(0.56 - 1.36)	0.95	(0.60 - 1.48)	0.84	(0.53 - 1.35)	1.02	(0.64 - 1.63)
	**3**	**2.13**	**(1.41 - 3.20)**	1.42	(0.93 - 2.15)	1.38	(0.90 - 2.10)	1.51	(0.98 - 2.32)	1.36	(0.87 - 2.13)	1.57	(1.00 - 2.48)
	**4 (low)**	1.00		1.00		1.00		1.00		1.00		1.00	
**Location**^**b**^	**Private hospital**	**0.08**	**(0.04 - 0.16)**	**0.17**	**(0.08 - 0.36)**	**0.19**	**(0.08 - 0.42)**	n/a		**0.19**	**(0.09 - 0.41)**	**0.20**	**(0.09 - 0.43)**
	**RH-MR**^**b**^	0.78	(0.11 - 5.61)	0.57	(0.08 - 4.13)	0.57	(0.08 - 4.12)	0.58	(0.08 - 4.18)	0.64	(0.09 - 4.66)	0.42	(0.06 - 3.07)
	**RH-RR**^**b**^	**2.66**	**(1.93 - 3.65)**	**1.51**	**(1.04 - 2.18)**	**1.50**	**(1.03 - 2.18)**	**1.51**	**(1.05 - 2.19)**	**1.51**	**(1.01 - 2.27)**	1.12	(0.77 - 1.63)
	**MH-RR**^**b**^	0.73	(0.45 - 1.18)	**0.46**	**(0.28 - 0.77)**	**0.50**	**(0.30 - 0.84)**	**0.46**	**(0.27 - 0.77)**	**0.46**	**(0.25 - 0.82)**	**0.45**	**(0.25 - 0.80)**
	**MH-MR**^**b**^	1.00		1.00		1.00		1.00		1.00		1.00	
**IHD type**	**MI**	**2.35**	**(1.63 - 3.39)**	0.89	(0.59 - 1.35)	0.86	(0.57 - 1.30)	0.92	(0.60 - 1.39)	0.92	(0.59 - 1.45)	1.07	(0.69 - 1.64)
	**Unstable Angina**	**4.05**	**(2.83 - 5.79)**	**1.97**	**(1.34 - 2.89)**	**1.87**	**(1.27 - 2.76)**	**1.97**	**(1.33 - 2.92)**	**1.92**	**(1.26 - 2.93)**	**2.12**	**(1.44 - 3.12)**
	**Other IHD**	1.00		1.00		1.00		1.00		1.00		1.00	
**Admission type**	**Emergency**	**14.9**	**(7.64 - 29.07)**	**5.93**	**(2.88 -12.20)**	n/a		**5.39**	**(2.53 -11.60)**	**6.91**	**(3.05 - 15.66)**	**7.01**	**(3.26 - 15.05)**
	**Booked**	1.00		1.00				1.00		1.00		1.00	
**Co-morbidity**	**Charlson index**	1.04	(0.97 - 1.12)	1.00	(0.93 - 1.09)	0.99	(0.91 - 1.08)	1.00	(0.93 - 1.09)	0.98	(0.89 - 1.08)	0.97	(0.89 - 1.06)
**Alc/MH**^**c**^	**Alcohol only**	**7.27**	**(5.14 - 10.29)**	**2.92**	**(2.01 - 4.24)**	**2.97**	**(2.03 - 4.33)**	**2.93**	**(2.02 - 4.26)**	**2.68**	**(1.75 - 4.11)**	**2.77**	**(1.86 - 4.12)**
	**MH only**	1.76	(0.9 - 3.46)	1.42	(0.72 - 2.83)	1.48	(0.75 - 2.95)	1.30	(0.63 - 2.69)	1.16	(0.51 - 2.66)	1.49	(0.75 - 2.98)
	**Both**	**7.87**	**(4.42 - 14)**	**2.97**	**(1.62 - 5.47)**	**3.13**	**(1.7 - 5.77)**	**2.99**	**(1.63 - 5.51)**	**3.21**	**(1.52 - 6.80)**	**2.04**	**(1.00 - 4.18)**
	**Neither**	1.00		1.00		1.00		1.00		1.00		1.00	
**Admission period**	**2000-2004**	0.92	(0.70 - 1.20)	0.90	(0.68 - 1.18)	0.95	(0.72 - 1.26)	0.91	(0.69 - 1.2)	0.89	(0.66 - 1.19)	0.96	(0.72 - 1.28)
	**2005-2008**	1.00		1.00		1.00		1.00		1.00		1.00	

Adj OR: odds ratio adjusting for all variables shown table; 95% CI: 95% confidence interval. Bold ORs are statistically significant.

^**a**^ Social Disadvantage.

^**b**^ Hospital type in combination with residential location: RH=Rural hospital; MH= Metro hospital; MR=Metro Residence; RR=Rural Residence.

^**c**^ History of alcohol and/or mental health admission.

Emergency admission (OR=5.9, 95% CI 2.9-12.2), alcohol with or without mental health-related admission history (alcohol-related OR=2.9, 95% CI 2.0-4.2) and Aboriginality (OR 2.3, 95% CI 1.5-3.5) remained the strongest predictors of DAMA in the multivariate model, with social disadvantage, Charlson score and calendar period the only variables not significant (Table 2). The models using different definitions of Aboriginality gave similar results to the main model, with the OR for Aboriginality being 2.5 when using the Aboriginal flag on the index admission only compared with 2.3 for Aboriginal flags defined by any (current or previous) admission as well as by at least 25% of all admissions.

There was consistency in the ORs in the different multivariate models for the restricted sub-cohorts. In these models, Aboriginal patients were between 2.0 and 2.4 times more likely to DAMA. Likewise, the ORs were similar across cohorts for males (ORs 2.1-2.2), UA compared with other IHD (ORs 1.9-2.1), emergency admissions (ORs 5.4-7.0), history of alcohol-related admissions (ORs 2.7-3.0), age (ORs 0.96-0.97) and rural hospital and residence compared with metropolitan hospital and residence (OR 1.1-1.5).

No interactions between Aboriginality and other variables were identified on formal testing. However, in the stratified analysis (Table 3) the ORs for sex and emergency admission were significant in non-Aboriginal patients only, whilst history of alcohol-related admissions was a significant predictor in both groups. In terms of clinical variables, Aboriginal patients were more likely to DAMA if they had fewer comorbidities (borderline significance, with the odds of DAMA decreasing by 17% for each unit increase in Charlson score) but no association was seen in non-Aboriginal patients. In non-Aboriginal patients, there was a higher odds of DAMA for UA than for MI, whereas the type of IHD diagnosis had minimal impact on DAMA in Aboriginal patients. However, this may have been due to the small number of events given the wide 95% confidence intervals in the Aboriginal group.

**Table 3:** Odds Ratios (OR) for discharge against medical advice during first-ever hospital episode for ischaemic heart disease, stratified by Aboriginality, Western Australia 2000-2008

		**Aboriginal**		**Non-aboriginal**	
		**OR (95% CI)**		**OR (95% CI)**	
Sex	Male	1.27	(0.67 - 2.39)	**2.50**	**(1.64 - 3.82)**
	Female	1.00		1.00	
Age		0.96	(0.93 - 0.99)	0.97	(0.95 - 0.98)
Social disadvantage	1 (high)	1.07	(0.29 - 3.89)	1.24	(0.79 - 1.94)
	2	1.00	(0.26 - 3.84)	0.87	(0.54 - 1.4)
	3	0.86	(0.2 - 3.72)	1.51	(0.97 - 2.33)
	4 (low)	1.00		1.00	
Hospital type in combination with residential location	Private hospital	0.00		**0.17**	**(0.08 - 0.37)**
	Rural hospital, metro residence	0.00		0.70	(0.1 - 5.08)
	Rural hospital, rural residence	1.96	(0.97 - 3.98)	1.39	(0.89 - 2.19)
	Metro hospital, rural residence	**0.37**	**(0.15 - 0.89)**	0.55	(0.29 - 1.03)
	Metro hospital, metro residence	1.00		1.00	
IHD diagnosis type	Other IHD	0.51	(0.21 – 1.20)	1.36	(0.85 - 2.18)
	Unstable Angina	0.95	(0.46 – 1.98)	**2.71**	**(1.89 – 3.87)**
	Myocardial infarct	1.00		1.00	
Admission type	Emergency admission	4.72	(0.62 - 36.16)	**6.24**	**(2.86 - 13.6)**
	Booked admission	1.00		1.00	
Co-morbidity	Charlson score	**0.83**	**(0.69 - 1)**	1.05	(0.97 - 1.15)
History of admission for alcohol or mental health related issue	Alcohol only	**2.44**	**(1.3 - 4.56)**	**3.06**	**(1.94 - 4.83)**
	Mental health only	n/a		1.71	(0.85 - 3.41)
	Both	1.88	(0.61 - 5.83)	**3.70**	**(1.82 - 7.5)**
	Neither	1.00		1.00	
Admission period	2000-2004	0.82	(0.46 - 1.49)	0.90	(0.66 - 1.22)
	2005-2008	1.00		1.00	

OR: odds ratio; 95% CI: 95% confidence interval.

Aboriginal R-Square=0.153; Non- Aboriginal R-Square=0.138.

## Discussion

This is the first detailed person-based study of determinants of DAMA focussing on Australian patients with IHD. The study shows that the known risk factors for DAMA – age, sex, emergency admission and history of alcohol-related admissions – also apply to this particular clinical group. Patients living in rural areas while attending a non-metropolitan hospital had a 50% higher odds than metropolitan patients who attended metropolitan hospitals. Even after adjustment for all these factors, the risk of DAMA in Aboriginal IHD patients remained more than double that of non-Aboriginal patients (down from a crude odds ratio of >8), with some risk factors for DAMA being consistent between Aboriginal and non-Aboriginal patients. However, unlike non-Aboriginal patients, the odds of DAMA in Aboriginal patients was not associated with severity of event (UA versus MI). Additionally, Aboriginal males and females had similar risks of DAMA. These results were not sensitive to the way we identified Aboriginal status in the administrative data.

Since DAMA patients have poorer outcomes, [2,6] understanding the risks and reasons for DAMA are important for both health system policy and practical clinical management in the hospital, regardless of the absolute number of DAMA cases involved. Drug and alcohol dependency is one of the most frequently cited factors associated with DAMA [6] and in the current study was a strong predictor in both Aboriginal and non-Aboriginal patients. Our finding of a higher risk of DAMA among emergency admissions may reflect the unexpected nature of the admission [22]. Additionally, the inability to attend to family, social and other obligations or needs puts pressure on patients who respond by self-discharge [6,23]. A study examining whether and why DAMA varies by ethnicity suggested that *where* patients are admitted, rather than individual factors, contribute to disparities in DAMA in the American setting [24]. Thus the higher risk in rural Australian hospitals may reflect lower confidence of patients in smaller hospitals, poorer responses from hospital staff who may have less experience, fewer resources, lower skills and/or be overworked, less anonymity for patients in small rural centres, and a sense of less serious illness if transfer is not required. International medical graduates are over-represented in Australian rural hospitals and often have limited orientation, education and training in working with Aboriginal patients, potentially resulting in Aboriginal patients feeling unwelcome and misunderstood [25,26].

The higher DAMA risk in Aboriginal heart patients indicate that there are some unique personal and system factors driving DAMA in this group [23]. There is evidence from early studies that patient anxiety and anger is associated with self-discharge, [6] with poor professional-patient and inter-professional communication also playing a major role in patients’ perceptions and experiences [22]. The discomfort that many Aboriginal people experience in hospital [27] partly reflects persisting negative associations with hospitals and mainstream institutions [28]. As one researcher writes, “[c]ontemporary Aboriginal perspectives of hospitals continue to be shaped by the effect of colonisation, creating a depth of fear and anxiety that is difficult for non-Aboriginal people to comprehend.” [29] It has been argued that high DAMA rates reflect an underlying poor response from hospital staff to the needs of Aboriginal patients, with many services failing to recognise and acknowledge their special needs [11,23]. This negative association with health services is also seen through delayed presentation [30,31] and suboptimal uptake and adherence with treatments [32,33].

This study has a number of strengths over previous published studies of DAMA in the Australian context. The use of linked data allowed a person-based analysis (where we investigated a comparable event for all patients (their first-ever admission specifically for IHD) and only counted each person once at their discharge, even when they were transferred between hospitals. By focusing on IHD, a serious and potentially life-threatening condition, we have been able to investigate both demographic and clinical variables as risk factors for DAMA. We were also able to ascertain histories of co-morbidity, alcohol-related events and previous DAMAs. We used three different methods for Aboriginal identification and demonstrated that the relationship between Aboriginality and DAMA was not sensitive to these.

Administrative data limit the type and quality of information available for analysis. Data are entered by trained clinical coders at each hospital from discharge summaries prepared by the discharging doctor, as well as from medical notes according to the Australian ICD coding standards manual. In addition, the Data Linkage Branch has its own internal data integrity and checking procedures for the various administrative datasets. Although we were unable to establish the reason for and circumstances surrounding the DAMA, we expect its coding to be accurate, reflecting strict hospital procedures for documenting DAMA for medico-legal reasons and due to the risk to patients. As neither residential address nor hospital identity was available to us (for confidentiality considerations) the distance between a patient’s residence and the hospital could not be considered more closely. Due to small numbers and the numerous hospital-residence combinations possible, the hospital-residence variable was crude. Social disadvantage scores were based on postcodes, and do not necessarily reflect individual-level disadvantage. Since the study did not include emergency department records, “did-not-wait” patients were not included. Additionally, the reliance on WA only meant that there is potential for misclassification regarding first-ever status as well as comorbidities and prior alcohol-related admissions. This is likely to have had minimal impact on the main results.

Identification of risk factors for DAMA events will assist in understanding their determinants and in designing interventions to reduce them. Various general strategies have been suggested [6]. High on the list are early recognition of patient discomfort (or lack of engagement) and identification of patients’ concerns/issues [23]. Pre-emptively addressing such issues can be challenging if systemic structures and/or staff behaviours contribute to patients’ discomfort [34] – discrimination and racism is often unreported and unchallenged [35], and may not be visible to insiders. Accordingly, hospitals need to reflect on service characteristics as well as patient factors that underlie DAMAs.

Related to this is the recommendation to screen for and deal with substance dependency proactively in an empathetic, even-handed way. However, we acknowledge that it may be difficult to manage patients with dependencies. Thus, a clear protocol for evaluating and intervening is required to support the alcohol-dependent patient, as is training staff in how to communicate and deal with such situations in a culturally appropriate way. Another strategy suggested is appointment of a staff member as patient advocate to help address the patient’s fears, complaints and concerns. This was effective in decreasing DAMAs by 30% in psychiatric patients [36]. In the Australian context, employing an Aboriginal Health Worker in a cardiology ward meant that Aboriginal inpatients had psychosocial and communication support and were less distressed which significantly reduced DAMAs [29]. A similar benefit was shown in Aboriginal medical inpatients in Alice Springs Hospital [37].

This study highlights the need for greater recruitment and retention of Aboriginal people in the health workforce, including interpreters for Aboriginal languages [23]. Cultural safety measures also need to be implemented at the staff-level in the hospital system. Additionally, post-discharge interventions should form an integral part of the management of DAMA patients with clear guidelines to contact the primary care providers, actively follow up patients [6] and engage families. Additional critical issues to be addressed in hospital care for Aboriginal cardiac patients were raised at a recent conference focused on Indigenous cardiovascular health, namely: addressing systemic racism; reconfiguring models of care to address the needs of Indigenous people; improving information systems and facilitating communication across the health care sector and with Indigenous communities [38].

## Conclusions

DAMA is embedded in the Aboriginal and Torres Strait Islander Health Performance Framework as an indirect indicator of the responsiveness of hospital services and the extent to which people vote ‘with their feet’. The Framework calls for routine evaluation of the hospital experiences of Aboriginal people, trialling of innovative programs and replication of successful ones. Effective interventions to reduce DAMA will benefit not only Aboriginal patients but almost certainly will improve the experience for all patients admitted to Australian hospitals. This is a worthy goal and the evidence reported in this paper suggests particular attention to improved approaches should occur especially in rural hospitals and be directed towards younger patients, males, Aboriginal people and those with alcohol and mental health problems.

## Abbreviations

ARIA: Accessibility/remoteness index of Australia; DAMA: Discharge against medical advice; ICD: International classification of diseases; IHD: Ischaemic heart disease; MI: Myocardial infarction; SES: Socio-economic status; UA: Unstable angina; WA: Western Australia.

## Competing interests

The authors have no competing interests to declare.

## Authors’ contributions

JK conceptualised the study, extracted and analysed the data and was main contributor to the write-up. MK contributed substantially to the statistical analysis and data interpretation, and reviewed the manuscript. FS and MH advised on study design, data extraction and integrity, and reviewed the manuscript. DB and AD assisted with the interpretation of findings and write-up of the manuscript. ST prompted interest in the original topic, assisted with the interpretation of the data and reviewed the manuscript. All authors read and approved the final manuscript.

## Pre-publication history

The pre-publication history for this paper can be accessed here:

http://www.biomedcentral.com/1472-6963/13/330/prepub
